# Supplementary material for: ‘It's all about patient safety’: an ethnographic study of how pharmacy staff construct medicines safety in the context of polypharmacy
Source: BMJ Open. 2021 Feb 5;11(2):e042504. doi: 10.1136/bmjopen-2020-042504 (PMC7925910; doi:10.1136/bmjopen-2020-042504)
Supplement: Supplementary data [file bmjopen-2020-042504supp001.pdf]

Staff Interviews (Pharmacy)

The focus of our research is on patients who are prescribed many medicines for their medical conditions (*polypharmacy*). In this interview we would like to **learn about the work you do** and about **who** and **what** is involved.

So first could you please start off by telling me:

- **how long you have worked here**
- **your job title and**
- **any training you've done to work here**

1. **What are the key routines – all the different things you have to do - you are involved in that support patients who are prescribed many medicines?**

2. **Please can you select ONE of these routines and talk me through what happens in detail** (*If they struggle to identify a routine we pick one e.g. 'dossette box production' 'repeat dispensing' 'checking routines' 'dossette box preparation' 'deblistering', ordering drugs, 'filling the robot')*
    - (What is involved in this routine?)
    - (Who is involved in this routine?)
    - Who is responsible for different aspects of this routine?
    - Can you describe a challenge that you have recently encountered while doing this particular work? How did you approach this?

3. **Please would you tell me about the example that you thought about before the interview?**
   (Pre-interview: Please would you come to the interview prepared to tell us about a particular story or incident, case, prescription or puzzle that has involved a patient who is prescribed many medicines)

    - Please will you talk me through it?
    - Why did you select this particular example?
    - (What happened / What happened next?)
    - How did this experience make you feel?
    - What did you learn from this experience?

4. **What are your main priorities *when managing the prescriptions of people who are prescribed many medicines* / *when you do your work?***
    - Why is this priority (are these priorities) important to you?
    - How does this priority influence what you do in practice?
    - Can you describe an example of something you have done recently in your work that shows your commitment to this priority?

5. **How has the electronic prescribing system changed how you work and what you have to do?**

6. **Is there something else you would like to talk about?**
